# Standardization of BCR-ABL1 p210 Monitoring: From Nested to Digital PCR

**DOI:** 10.3390/cancers12113287

**Published:** 2020-11-06

**Authors:** Aleksandar Jovanovski, Jessica Petiti, Emilia Giugliano, Enrico Marco Gottardi, Giuseppe Saglio, Daniela Cilloni, Carmen Fava

**Affiliations:** 1 Department of Clinical and Biological Sciences, University of Turin, 10043 Turin, Italy; giuseppe.saglio@unito.it (G.S.); daniela.cilloni@unito.it (D.C.); carmen.fava@unito.it (C.F.); 2 Division of Internal Medicine and Hematology, San Luigi Gonzaga Hospital, Orbassano, 10043 Turin, Italy; e.giugliano@sanluigi.piemonte.it (E.G.); enricogottardi@libero.it (E.M.G.)

**Keywords:** BCR-ABL1, Chronic myeloid leukemia, molecular monitoring, international scale, conversion factor, MMR

## Abstract

**Simple Summary:**

The diagnostic and clinical success of standardization of BCR-ABL1 p210 monitoring in chronic myeloid leukemia patients could be seen as a good example for further standardization of molecular monitoring in other gene rearrangements. In this review, we present the chronology of more than 20 years of scientific work that allowed to achieve this result. We believe that this paper will be useful for researchers who approach the chronic myeloid leukemia field.

**Abstract:**

The introduction of tyrosine kinase inhibitors in 2001 as a targeted anticancer therapy has significantly improved the quality of life and survival of patients with chronic myeloid leukemia. At the same time, with the introduction of tyrosine kinase inhibitors, the need for precise monitoring of the molecular response to therapy has emerged. Starting with a qualitative polymerase chain reaction, followed by the introduction of a quantitative polymerase chain reaction to determine the exact quantity of the transcript of interest-p210 BCR-ABL1, molecular monitoring in patients with chronic myeloid leukemia was internationally standardized. This enabled precise monitoring of the therapeutic response, unification of therapeutic protocols, and comparison of results between different laboratories. This review aims to summarize the steps in the diagnosis and molecular monitoring of p210 BCR-ABL1, as well as to consider the possible future application of a more sophisticated method such as digital polymerase chain reaction.

## 1. Introduction

Chronic myeloid leukemia (CML) is a myeloproliferative neoplasm with an increased proliferation and accumulation of leukocytes and precursor myeloid cells in bone marrow (BM) and peripheral blood (PB) with an annual age-adjusted incidence of 1.6 per 100,000 population [1]. CML is mainly characterized by t (9; 22) (q34; q11) chromosomal translocation [2], giving rise to BCR-ABL1 p210 fusion protein with constitutive activation of tyrosine kinase activity. In most CML patients (~95%), the BCR-ABL1 rearrangement arises from two major breakpoints, involving exons 13 or 14 of BCR and exon 2 of ABL1 (e13a2 and e14a2) [2].

In the beginning, CML patients were treated with chemotherapy agents such as cytarabine, hydroxyurea, and immunomodulatory agents like interferon-alpha. Nowadays, the standard of care is tyrosine kinase inhibitors (TKIs), which considerably improved response rates, survival, and quality of life of patients [3].

Cytogenetic analysis or fluorescence in situ hybridization (FISH) are important at diagnosis and until a Complete Cytogenetic Remission (CCyR) is achieved. After the introduction of TKIs, most patients reach a deep molecular response (DMR) that far exceeds the sensitivity of a cytogenetic evaluation [4,5,6]. In this scenario, BCR-ABL1 mRNA levels has become the most important molecular marker for the minimal residual disease (MRD) evaluation, defining the level of molecular remission and guiding clinical decisions, such as therapy changes or discontinuation [7]. Thus, the accurate monitoring of the BCR-ABL1 fusion transcript appears mandatory to follow TKIs-treated CML patients.

The modern idea of molecular monitoring in patients with CML was introduced 20 years ago when the process of international standardization started. It is impossible to separate the standardization of molecular monitoring process and the introduction of TKI therapy in CML patients. Together, these activities have created complex pathways of inter- and intra-laboratory collaboration with the fundamental aim of optimizing and equalizing criteria for diagnosis and clinical interpretation of the results.

Herein, we retraced the steps that allowed the standardization of BCR-ABL1 monitoring from qualitative to quantitative methods.

## 2. Nested PCR

The first phase of BCR-ABL1 monitoring was represented by the use of a qualitative assay: the nested PCR. This technique forecast two different primer sets, used in two subsequent amplification steps [8]. This evolution of conventional PCR has led to an increase in the sensitivity and specificity of the results. Monitoring of MRD through nested PCR allowed to stratify childhood acute lymphoblastic leukemia patients in several MRD-based groups, useful to choose the proper treatment protocol [9]. In the 90s, the European BIOMED-1 program harmonized protocols for MRD monitoring of several fusion transcripts, including BCR-ABL1, whose standardization was coordinated by Gabert’s group [10]. Fourteen European laboratories from eight different countries participated in the BIOMED-1 program with the aim to produce more comparable results for multicenter international treatment protocols.

The first goal of the program was to conform the reverse transcription (RT-PCR) method. Several procedures were compared taking into account the amount of RNA, the type of primers, the concentrations of reagents, the final volume of reaction, the enzyme type, and the temperatures and the incubation time for a common protocol [10].

Subsequently, the BIOMED-1 group focused on amplification procedures.

Concerning BCR-ABL1 evaluation, five different primers sets were designed, selected, and subsequently validated by all participating laboratories. The sensitivity achieved with only one PCR step ranged between 10^−3^ and 10^−4^, increasing to 10^−5^/10^−6^ using nested PCR [10]. These collaborative studies have resulted in a standardized RT-PCR protocol and PCR primer sets useful for MRD-monitoring worldwide.

At the same time, rapid technological development paved the way for the introduction of increasingly accurate technologies.

## 3. Quantitative Real-Time PCR

During the years, MRD monitoring has become increasingly important: it helps to predict treatment resistance and guide the course of treatment. It was clear the need to overcome the limits of a qualitative measurement, to move on to quantitative monitoring of BCR-ABL1 transcript with an aim to more favorable follow of the course of the disease.

In contrast with conventional PCR, in which the amplicon is detected by an end-point analysis, quantitative Real-Time PCR (qRT-PCR) allows analyzing the amplification during the exponential phase of the reaction using fluorescent molecules [11]. The cycle threshold (Ct) represents the cycle number when the fluorescence of a specific PCR product can be detected above the background signal. Ct values are inversely proportional to the amount of target nucleic acid in the sample. qRT-PCR also allows to obtain an absolute quantification: the initial amount of evaluated transcript can be calculated by comparing the Ct value with a standard curve generated with different known copies of target DNA [12,13].

With the introduction of qRT-PCR in routine practice, the BIOMED-1 program initiated a large study group with the purpose to standardize procedures for MRD monitoring [10].

### 3.1. Selection of Control Genes

One of the most important issues encountered with the introduction of qRT-PCR was the choice of a control gene (CG). A suitable CG can be defined as a gene with a stable expression, not affected by any experimental conditions, and should not show any pseudogenes [14,15,16]. In the early 2000s, the Europe Against Cancer (EAC) program started the first collaborative study aimed at selecting and validating a suitable CG to monitor BCR-ABL1 transcript by qRT-PCR [17]. In this study, all members of the network used the same sequence detection system and the same procedures; samples and plasmids were centrally prepared and distributed to the laboratories. Fourteen potential CGs were evaluated and, among these, only three were selected for a deep analysis: Abelson (ABL1), beta-2-microglobulin (B2M), and glucuronidase beta (GUSB). The EAC did not evaluate the breakpoint cluster region protein (BCR), already used and validated by several groups [18]. Beillard et al. identified the ABL1 gene as the most reliable CG because it was similarly expressed in normal and pathological samples and its correlation with BCR-ABL1 expression was the highest observed compared to B2M and GUSB. Nevertheless, a slight inaccuracy in measurement was highlighted of BCR-ABL1/ABL1 ratio at a high level of fusion transcripts, due to the co-amplification of ABL1 and BCR-ABL1 [17]. In the same year, Gabert et al. demonstrated that using a CG to normalize results greatly improved the data reproducibility between laboratories [19]. Furthermore, they established the reference ranges for the CGs in normal PB, BM, and PB stem cells [19]. Successively, Rulcova et al. [20] performed an independent analysis of the suitability of ABL1, B2M, and GUSB as CG to monitor CML patients. In contrast with Beillard et al. [17], they highlighted several perplexities about the use of ABL1, while they identify B2M as better CG [20]. Indeed, they confirmed and underlined that BCR-ABL1/ABL1 results were not linear when BCR-ABL1 levels are over 10%, probably because EAC primers also amplify BCR-ABL1. Although this criticism was already described and shared by the CML international community [17,21], it did not find a large consensus, because a precise quantification of BCR-ABL1 levels over 10% had poor clinical relevance.

In recent years, different papers highlighted that the time of declining of BCR-ABL1 transcript can result in distinctly different prognostic subgroups, prompting the implementation of dynamical parameters and the use of an alternative control gene [22,23]. Recently, Moisoiu et al. suggested the use of a mathematical transformation to mitigate this situation, continuing to use ABL1 as CG. Indeed, they propose to use a correction that considers the distinction between ratio and proportion. This correction can be applied to all the range of BCR-ABL1/ABL1, but it results as significant only when this percentage is over 10%. Using this strategy, ABL1 can be used as CG also when BCR/ABL1 IS values are > 10%, returning correct results [24].

### 3.2. International Standardization of p210 qRT-PCR Results

In the early 2000s, the International Randomized Study of Interferon versus STI571 (IRIS study) was conducted [18] to test imatinib versus interferon-cytarabine. After 12 months of treatment, imatinib has shown preponderance efficacy and significantly higher rates of transcript reduction in patients who had achieved complete cytogenetic remission [18]. It was also found that patients who had obtained at least 3-log reduction from the initial BCR-ABL1 transcript level had a likelihood of progression-free survival of 100% in the next 24 months [18]. Moreover, the 3-log reduction from the initial transcript level was defined as a major molecular response (MMR) [18].

During this trial, qRT-PCR, the gold standard for molecular diagnosis, was performed in three reference laboratories: Adelaide, London, and Seattle. The CG of choice in each lab was BCR and the results were expressed as a percentage of BCR-ABL1/BCR.

Due to the huge heterogeneity in the qRT-PCR steps among the three reference laboratories, it was observed a significant inter-laboratories difference in the reproducibility of both the pathological and control genes. Thus, the main problem was the interpretation of the differences between the obtained results.

To overpass this problem, the leading scientists in the IRIS study established that each reference laboratory should create its own assortment of the same 30 newly diagnosed untreated CML patients to extrapolate median value. This laboratory-specific median value was assumed as a standardized baseline, corresponding to 100% BCR-ABL1/ABL1. The 3-log reduction was defined as a reduction from the laboratory-specific median value and not as a log reduction calculated from the BCR-ABL1 level at the time of diagnosis for every newly diagnosed patient. In this way, the results of the three reference laboratories were normalized towards the standardized baseline.

**Figure 1:**
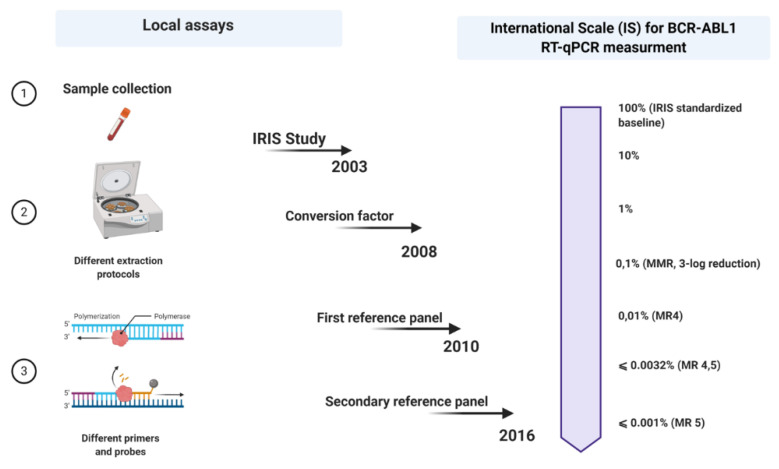
Schematic outline of all steps in the standardization process of p210 BCR-ABL1 transcript monitoring in patients with chronic myeloid leukemia (CML). Each laboratory uses its local assay for quantitative qRT-PCR and then expresses its results on an international scale (IS) using a conversion factor (CF) based on a reference panel (the last reference panel from 2016). Picture created with Biorender.com.

A few years later, in 2005, an international consensus meeting was held in Bethesda, USA [25]. This can be seen as a milestone point associated with the expression of BCR-ABL1 measurements on an international scale (IS). Regarding this, when the values of the BCR-ABL1 transcripts produced anywhere worldwide are expressed on an IS, they could be more likely comparable. However so far, there were only two criteria on which the IS could be fastened: the definition of standardized baseline and MMR (Figure 1).

Meanwhile, the 30 samples used during the IRIS study were no longer available, so a new reasonable replacement had to be found. In favor of that, two laboratories, one from Germany and the other from Australia, exchanged RNA and cDNA samples, reproducing the standardized baseline using the ABL1 as a control gene [26]. The BCR-ABL1/ABL1 ratio obtained in the German laboratory among the patients who have achieved MMR was 0.12%, corresponding with the 3-log reduction from the standardized baseline established in the IRIS trial, fixed at 0.10% (MMR) [25]. The level of 1% approximately corresponds to the achievement of CCyR [25].

### 3.3. The “Era” of Conversion Factor

The definition of standardized baseline and MMR paved the way for expressing the results in a standardized international numerical scale (IS), more practical than having results expressed as “log reduction”. Although the idea of an IS seemed revolutionary, its implementation proved to be a major challenge. Differences in the methods adopted by various laboratories determined great variability in the results obtained. To face this problem, the EAC group had previously started a standardization of the qRT-PCR protocol. Despite their efforts to reduce variability, significant differences between results were still present [19].

The calculation of a laboratory-specific conversion factor (CF) was intended to equalize these discrepancies. Branford et al. explained the first attempt to establish validated CFs [27]. In a big collaborative study, Adelaide was the reference laboratory, and 39 satellite laboratories participated in this project. The Bland and Altman method was used to calculate a CF for each lab, comparing the dataset from the same samples generated by the participant laboratories with that obtained by the reference laboratory (Figure 1).

The problem with CFs was not just about adjusting the results between reference laboratory and test laboratories. In fact, a time-consuming periodic validation was required, that demanded an additional exchange of samples. Moreover, before any exchange of samples, the whole process of molecular diagnosis must be optimized, because if a single constituent of the diagnostic step changes, the previous laboratory-specific CF is no longer applicable.

The standardization process is impossible to be performed worldwide if only one laboratory is taken as a reference. As a solution, at a meeting of the European Treatment and Outcome Study (EUTOS) group in 2009, the idea of a reference national or regional laboratory was discussed [28].

### 3.4. Development of Reference Panels and Commercial Kits

In favor of easier access to the IS, developing a reference panel and/or standard kit for molecular diagnosis would solve the problem of CF unsustainability over time. In 2010, the World Health Organization (WHO) developed the first International Genetic Reference Panel for the quantification of BCR-ABL1 mRNA [29]. Ten laboratories, all with pre-validated CFs, participated in this study, using the three most used control genes: ABL1, BCR, and GUSB. The creation of this reference panel is based on the preparation and titration of different dilutions of freeze-dried cell lines, such as HL-60, as BCR-ABL1 negative, and K562, as BCR-ABL1 positive. The material was prepared in 4-step dilutions (10%, 1%, 0.1%, and 0.01%) of K562 cells in the HL-60 cell line, considering that these dilutions are enough to provide more robust statistical results (regression line). About 3500 vials were produced for each level of dilution. Since the worldwide number of laboratories that diagnose patients with CML is around 500, the primary reference material is not sufficient to meet the full needs [29]. In fact, the primary reference material was reserved only to produce secondary reference reagents. Meanwhile, several secondary reference panels have been developed that allowed the expression of the results on IS [30,31,32].

In parallel with the improvement of the qRT-PCR method, the development of second- and third-generation TKIs also took place. As we have already discussed, MR is a strong predictive factor and a direct indicator of the effect of therapy. With the increase of the five-year survival rate among the CML patients and the number of patients achieving responses <0.1%, there was a need to determine different degrees of depth of response. The terms MR4, MR4.5, and MR5 have started to be used to indicate levels of disease that are ≤0.01% BCR-ABL IS (4-log reduction from IRIS baseline), ≤0.0032% BCR-ABL IS (4.5-log reduction from IRIS baseline), and ≤0.001% BCR-ABL IS (5-log reduction from IRIS baseline), respectively [33]. In addition, the number of copies of the control genes were also included to account for the sensitivity of the measures, even more important in cases with undetectable BCR-ABL1 transcripts.

More recently, Cross et al. developed a secondary reference panel which was cell-based and reproduced the first WHO panel with an additional level of disease MR4.5. In total, 44 laboratories participated in the study and to date, the secondary reference panel has been used as an indispensable part of the molecular monitoring of CML patients worldwide (Figure 1).

The standardization process never ends, but it is constantly evolving thanks to the application of new knowledge and new methodologies. In recent years, technological development has allowed a new technique to appear in the world of CML molecular monitoring: the digital PCR (dPCR).

## 4. Digital PCR

**Figure 2:**
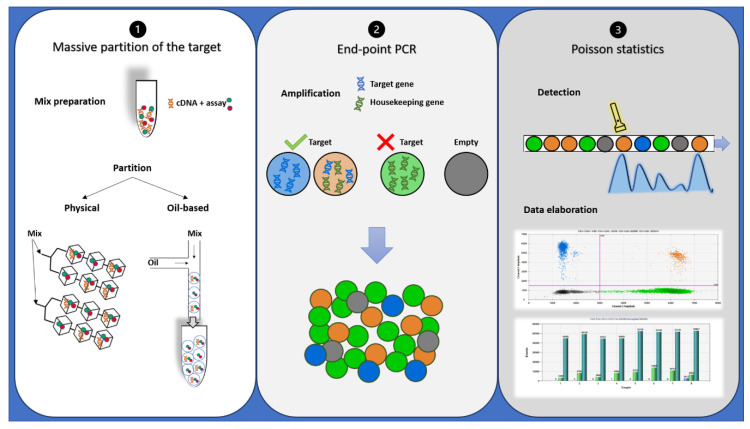
Description of droplet PCR workflow: (**1**) PCR reaction mixtures for each sample were partitioned in a microfluidic chip or through an oil-based emulsion. (**2**) The partitioned samples were placed into a standard thermal cycler for end-point PCR amplification: In the droplets containing target cDNA, the specific probe hydrolysis occurs and bright fluorescence appears, while in the droplets containing no target molecules (empty), only background probe fluorescence results. (**3**) Each droplet’s fluorescence was detected and processed into a two-dimensional scatter plot display. The number of droplets within each gate was then counted.

Despite oncologists started talking about dPCR in 1999 [34], it has only recently appeared to be a suitable alternative or completion for qRT-PCR in disease monitoring, especially for low levels of disease [35,36]. dPCR works with the same primer sets, fluorescent molecules, and reagents of qRT-PCR, but it is characterized by a massive partition of the sample into single PCR reactions [37]. It also allows performing an absolute quantification without the need for standard curves or reference material. In addition, dPCR results are evaluated by an end-point analysis, reducing error rates by removing the amplification efficiency dependence of qRT-PCR [38]. Indeed, dPCR is independent by primer efficiency, in respect to qRT-PCR, where primer efficiency has a huge impact on accurate quantification. dPCR workflow is based on three main steps: massive partition of the target, end-point PCR, and Poisson statistics (Figure 2) [39]. Currently, different commercialized platforms are available, principally divided into two categories: micro-well chip-based or microfluidic-chamber-based (cdPCR) and droplet dPCR (ddPCR) technologies. These platforms differ from the method used to partition the samples: cdPCR is based on a physical subdivision, while ddPCR is based on emulsion PCR technology [38].

### 4.1. dPCR to Monitor BCR-ABL1

With the introduction of the definition of MR4 and MR4.5 for the characterization of deep molecular response in CML patients, which is a prerequisite for a possible discontinuation of TKIs, the use of dPCR has become very attractive [40]. In recent years, several studies compared the use of dPCR and qRT-PCR to monitor BCR-ABL1 levels.

In a study conducted on 43 CML patients, Goh et al. demonstrated that dPCR could successfully detect BCR-ABL1 transcripts in qRT-PCR negative samples (24 out of 32) [41], suggesting the potential use of dPCR as highly sensitive approach in CML management. Successively, other groups demonstrated the relevance of dPCR in evaluating molecular response [42,43,44], especially for its ability to predict relapse after TKIs discontinuation [35,36,45].

In 2017, Alikian et al. [46] performed a comparative study on 102 samples to evaluate the performance of four different platforms: conventional qRT-PCR, QuantStudio 3D Digital PCR System (QS3D), QX200 droplet dPCR System, and RainDrop droplet dPCR System. Their findings suggest that qRT-PCR and dPCR systems have comparable sensitivity in identifying MR3 patients, while dPCR is superior for MR4 and MR4.5 levels. They found a very high correlation between qRT-PCR and all the dPCR systems in measuring BCR-ABL1, ABL1, and %BCR-ABL1/ABLIS. Moreover, they described some false positive samples for Raindrop and QS3D platforms, while only one positive droplet in 1 of the 30 negative controls was found using the QX200 system [46].

Another interesting advantage of dPCR concerns the ability to correctly evaluate the BCR-ABL1 e14a2 variant. Indeed, Kjaer et al. [47] identified a variant-specific discrepancy in the quantification of BCR-ABL1 e13a2 and e14a2 transcripts using the EAC assay, suggesting an underestimation of the e14a2 variant by qRT-PCR. In 2019, a study conducted on 142 CML patients demonstrated that there were no differences in the mean values of BCR-ABL1 obtained by dPCR between e13a2 and e14a2 patients. All this together supports the use of dPCR as an alternative to qRT-PCR in order to provide more accurate BCR-ABL1 monitoring in routine practice and long-term disease management [48].

Nowadays, dPCR has not undergone yet a standardization process. Chung et al. evaluated for the first time the performance of conventional qRT-PCR (Ipsogen BCR ABL1 Mbcr IS MMR RQ PCR assay) and QXDx BCR-ABL %IS ddPCR assay (Bio-Rad). They found Bio-Rad assay showed a very low coefficient of variation (<10%), high linearity (R2 = 0.998) and a very strong correlation (R2 = 0.996) with the qRT-PCR assay. In addition, no outliers and no false-positive results were observed using QXDx BCR-ABL %IS ddPCR assay. They then suggested that this ddPCR kit could be a reliable and promising tool for MRD monitoring in CML patients [49].

Subsequently, another multicenter Italian study compared the agreement between the measures, the repeatability, and the inter-laboratory reproducibility of QXDx BCR-ABL %IS ddPCR assay (Bio-Rad) and three different qRT-PCR kits for the quantification of BCR-ABL1. They concluded that ddPCR has a good agreement with qRT-PCR. Furthermore, they found smaller coefficients of repeatability of ddPCR for all levels of disease, which imply a slightly better precision compared to qRT-PCR [50]. More recently, the EAC group set up a multiplex ddPCR assay using the EAC primer/probe sets for BCR-ABL1 and ABL1 and compared the results with qRT-PCR and the QXDx BCR-ABL %IS ddPCR assay (Bio-Rad). They concluded that either ddPCR assays showed a shift to a higher MR level compared with qRT-PCR. Furthermore, they found that QXDx BCR-ABL %IS ddPCR assay (Bio-Rad) is more sensitive and specific for BCR-ABL1 compared to EAC ddPCR assay [51].

### 4.2. dPCR to Monitor BCR-ABL1 Atypical and Patient-Specific Transcripts

One to two percent of the CML patients showed breakpoints on chromosome 9 and 22 in uncommon points causing the arrangement of atypical (e13a3, e14a3, e19a2, e8a2, etc.) or patient-specific transcripts. Conventional qRT-PCR kits do not have the competence to amplify these sequences. Indeed, MRD monitoring for these patients is still performed only by cytogenetic, FISH analysis, and qualitative nested PCR [5], making it impossible to demonstrate the achievement of a MMR. This limitation decreases the prognostic information and automatically excludes patients showing atypical transcripts from the clinical protocol of treatment discontinuation [5,6]. Despite this, Dragani et al. described some patients with atypical transcripts who have discontinued TKIs treatment, successfully maintaining the clinical remission [52].

Since the dPCR allows the quantification of target molecules without the use of standard curves, showing a shorter standardization process, it seems to be very attractive to quantify the molecular response in patients with rare atypical fusion transcripts. In 2015, Zagaria et al. reported a case of blast-crisis CML patient with a rare e6a2 transcript. They demonstrated that ddPCR is a very useful technology in MRD monitoring when qRT-PCR assay is lacking [53]. Recently, Petiti et al. described a chronic-phase CML patient with a unique atypical transcript (BCR e13(-53nt)/ins 119nt ABL 1b/ABL ex2) where the designing of a patient-specific ddPCR assay allowed to quantify the MRD from diagnosis to follow up [54]. Finally, a preliminary study performed on 65 samples from 11 CML patients described two different ddPCR assays designed to monitor e13a3, e14a3 and e19a2 levels. The assays showed high linearity and a limit of detection of 0.001% BCR-ABL1/ABL1, providing a new method to monitor the MRD also in CML patients with atypical transcripts [55].

Further studies should be done to improve and standardize the MRD monitoring process of atypical transcripts. This information could improve disease treatment and include more patients in the clinical protocols of TKIs discontinuation.

## 5. Conclusions

CML is the first human cancer that was treated with target therapy, TKIs, and in this way, the molecular monitoring of treatment efficacy has become essential.

The successful clinical use of the standardized e13a2 and e14a2 fusion transcripts in monitoring of CML patients so far could be seen as good example for further standardization of molecular monitoring in other gene rearrangements. This should be considered not only in the onco-hematology field, but also in the other oncology diseases where specific gene rearrangements are presented.
